# Direct Experimental Evidence of Transient Au^δ+^ Oxide in Au Electrooxidation

**DOI:** 10.1021/jacs.5c13087

**Published:** 2026-02-25

**Authors:** Jesús Redondo, Ane Etxebarria, Pankaj Kumar Samal, Llorenç Albons, Roser Fernandez Climent, Sabine Auras, Břetislav Šmíd, Xiaohui Ju, Peter Matvija, Frederik Schiller, Martin Setvín, Josef Mysliveček, Sara Barja

**Affiliations:** † Department of Surface and Plasma Science, 37740 Charles University, Prague 180 00, Czech Republic; ‡ 16379 Centro de Física de Materiales CFM-MPC (CSIC-UPV/EHU), San Sebastián 20018, Spain; § Department of Polymers and Advanced Materials: Physics, Chemistry and Technology (PMAS), University of the Basque Country UPV/EHU 20018, Donostia-San Sebastián, Spain; ∥ Department of Chemistry and Biochemistry, Mendel University in Brno, Brno 613 00, Czech Republic; ⊥ Donostia International Physics Center, San Sebastián 20018, Spain; # Ikerbasque, Basque Foundation for Science, Bilbao 48009, Spain

## Abstract

Anodic electrooxidation of noble metals in acidic media represents a central topic in electrocatalysis and energy conversion due to its relevance for the oxygen evolution reaction (OER). In particular, gold electrodes are traditionally assumed to oxidize directly from the metallic state (Au^0^) to a trivalent state (Au^3+^) at high potentials relevant to OER. However, this long-standing paradigm has been increasingly challenged by recent in situ characterization studies. Here we report the direct observation of a metastable gold oxidation state (Au^δ+^, where 0 < δ < 3) preceding the formation of Au^3+^. This species is electrochemically induced on Au(111) in 0.1 M H_2_SO_4_ and observed at the electrode surface under ultrahigh vacuum (UHV) using quasi-in situ X-ray photoelectron spectroscopy (XPS). Au^δ+^ manifests a distinct chemical shift in XPS without conforming to a fully evolved Au^3+^ oxidation state. Atomic-resolution imaging via low-temperature scanning probe microscopy (LT-STM/nc-AFM) revealed that the Au^δ+^ phase forms an amorphous oxide layer. This study provides evidence of a two-step mechanism for Au electrooxidation accompanying the OER onset. Formation of the Au^δ+^ surface oxide precedes the formation of the bulk Au^3+^ oxide responsible for the OER. This mechanism strongly challenges the thermodynamically predicted one-step Au^0^ → Au^3+^ oxidation model. The two-step Au-oxidation mechanism parallels the proposed Pt electrooxidation pathway, suggesting a general oxidation mechanism for noble metals.

## Graphical abstract

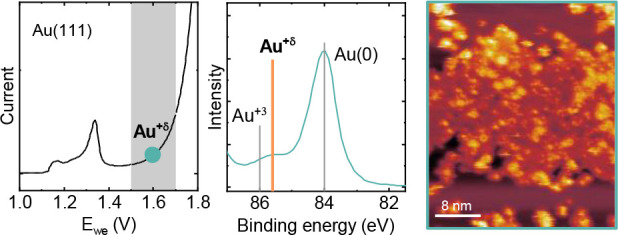

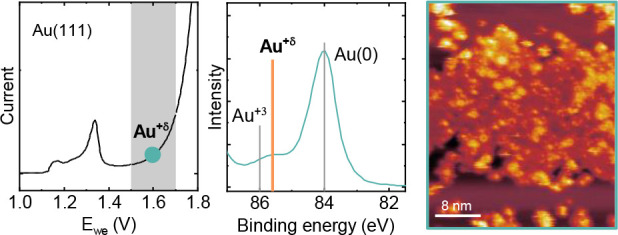

## Introduction

The chemical inertness of bulk gold (Au) renders this noble metal as an exceptional substrate for fundamental electrochemical studies,^1^ energy conversion reactions,^2,3^ and selective detection of a wide array of analytes.^4^ Au’s inherent resistance to corrosion^5^ and its weak interaction with target species under specific conditions make Au an ideal material for applications demanding a stable, nonreactive interface.

However, Au’s behavior in electrochemical environments is demonstrably more complex and dynamic. Under electrochemical conditions, the surface of gold electrodes undergoes significant changes, challenging their traditional perception as a purely inert material. Au surface transformation, often involving intricate formation and reconstruction of surface oxide phases, unveils diverse Au catalytic functionalities. Thereby, beyond its role as a simple substrate, gold demonstrates capacity as an active electrocatalyst in processes such as electrooxidation of biomass-derived molecules or alcohols (e.g., ethanol, glycerol),^6–8^ electrode for batteries,^9^ and water electrolysis.^10^ The transition from an inert, simple substrate to an active catalyst is governed by the complex formation and transformation of its surface oxide phases. This phenomenon elevates gold as an inherent precatalyst that undergoes significant reconstruction toward its active phase.

The critical role of these dynamic surface transformations has been formerly explored by cutting-edge in situ spectroscopic and microscopic techniques, including surface-enhanced Raman spectroscopy (SERS),^11^ electrochemical (EC) scanning tunneling microscopy (STM),^12^ operando X-ray absorption spectroscopy (XAS),^13^ high-energy-resolution fluorescence-detected X-ray absorption near-edge structure (HERFD-XANES),^14^ and X-ray photoemission spectroscopy (XPS).^15^ A central gap in prior work largely reflects a prevailing Au^0^/Au^3+^ interpretive bias (consistent with one-step Au^0^ → Au^3+^ mechanism) inferred primarily from difference in XPS core level shifts,^16,17^ with Au^3+^ oxidation state typically treated as the thermodynamically favored end point.^10^ The proposed one-step oxidation mechanism is furthermore being challenged by electrochemical capacitance measurements which infer an initial adsorption of hydroxyl species (Au–OH) and subsequent formation of Au_2_O, and AuO species,^18,19^ as well as by recent advances in operando surface-science instrumentation, where an Au^1+^-like contribution has been explicitly noted as distinct from the Au^3+^ component.^15^ We frame the discussion in terms of one-step and two-step terminology as an operational distinction. One-step denotes continuous oxygen uptake on Au(111) without a spectroscopically resolvable surface intermediate; two-step denotes nucleation and saturation of a thin, surface-limited Au^δ+^ layer that precedes bulk-like Au^3+^ oxide growth.^1,11^

Importantly, the formation of Au oxide phases preceding the formation of Au^3+^ species and the oxygen evolution reaction (OER) have only been discussed in electrochemical environments. This behavior underscores the profound influence of the aqueous electrolyte and applied potential in stabilizing transient or low-oxidation-state gold oxide species, creating a significant knowledge gap in fully understanding the intrinsic chemical and structural properties of intermediate Au-oxidation phases outside reaction conditions.

In this study, we report unambiguous evidence of an intermediate Au^δ+^ (0 < δ < 3) surface oxide on Au(111), formed electrochemically in acidic media and explored via quasi-in situ XPS, and scanning probe microscopy (SPM) under ultrahigh vacuum (UHV) at low temperature (<4.8 K). Our results demonstrate the existence of a surface-limited Au^δ+^ state that is distinct from the Au^3+^ bulk-phase: (i) it exhibits a clearly different binding energy (B.E.) signature in XPS; (ii) it saturates at the surface without a continuous, monotonic evolution to Au^3+^ (consistent with a two-step model); and (iii) it is confined to a thin, amorphous surface film. The observed Au^δ+^ phase is metastable in UHV, spontaneously reducing to Au^0^ over time. The direct observation of this metastable transition state, outside of operando conditions, provides precise characterization supporting a two-step gold electrooxidation, thereby reconciling the discrepancies between thermodynamic considerations and observed oxidation pathways.

## Results

For exploring the potential-induced changes of Au(111) single crystal electrodes upon anodic polarization, we employed the combination of electrochemical techniques with surface chemical and atomic-scale structural characterization. We achieved this by employing a custom-designed transfer system that enables transfer of samples from an electrochemical (EC) cell to the UHV analysis chamber under inert atmosphere of Ar.^20–23^ This integrated setup is critical, as it prevents air exposure and effectively preserves the chemical and structural composition of the surface in its stationary state during transfer, allowing for experimental observation of transient surface species. A detailed schematic of the EC-UHV transfer setup and the methodological sequence is shown in Figure S1 and described in the Methods section and Supporting Information. The surface chemical state of the samples emersed from the EC cell was explored by XPS as a function of the applied potential. Similarly, atomic-scale structural characterization of the samples emersed from the electrolyte was performed using combined scanning tunneling and noncontact atomic force microscopy (STM/nc-AFM) qPlus sensors working in UHV at a cryogenic temperature (<4.8 K). This unique, comprehensive approach enables the integration of EC, chemical, and structural insights,^20,24,25^ providing a detailed understanding of the nature and stability of the electrochemically formed Au surface oxides.

Figure 1 shows the representative linear sweep voltammetry (LSV) and corresponding XPS spectra of Au(111) upon chronoamperometry (CA) measurements at potentials below and above the OER onset in 0.1 M H_2_SO_4_ (1.66 V vs Ag/AgCl). The OER onset is estimated by the intersection of the tangents from the current baseline and the rising slope of the anodic current,^11^ Figure S2. Unless otherwise stated, we refer all of the potentials to the Ag/AgCl reference electrode. The LSV profile in Figure 1a is characteristic of single-crystalline Au(111) electrodes in 0.1 M H_2_SO_4_. Two broad features below 1.34 V, labeled A and B in Figure 1a, are commonly assigned to surface-oxidation waves^17^ consistent with (i) initial OH adsorption/charge accumulation and (ii) onset of place-exchange that inserts O into the topmost Au layers, indicative of surface oxidation.^18,26^ These oxidative peaks are followed by a current plateau, which precludes the current increase due to O_2_ formation in the OER regime.^11^ Following each electrochemical treatment, the electrode was removed from the electrolyte under potential control, rinsed with Ar-purged Milli-Q water, and transferred through an inert atmosphere into UHV (see Methods and Figure S1). Figure 1b,c shows the Au 4f and O 1s XPS spectra, respectively, obtained after 30 min CA at 1.5 V (orange), 1.6 V (cyan), 1.7 V (dark blue), and 1.8 V (green). Dots of the same color mark the position of these potentials on the LSV in Figure 1a. Prior to each CA experiment, the Au(111) surface was UHV cleaned by cycles of sputtering and annealing and subsequent cyclic voltammetry (CV) conditioning, as shown in Figure S3.

**Figure 1:**
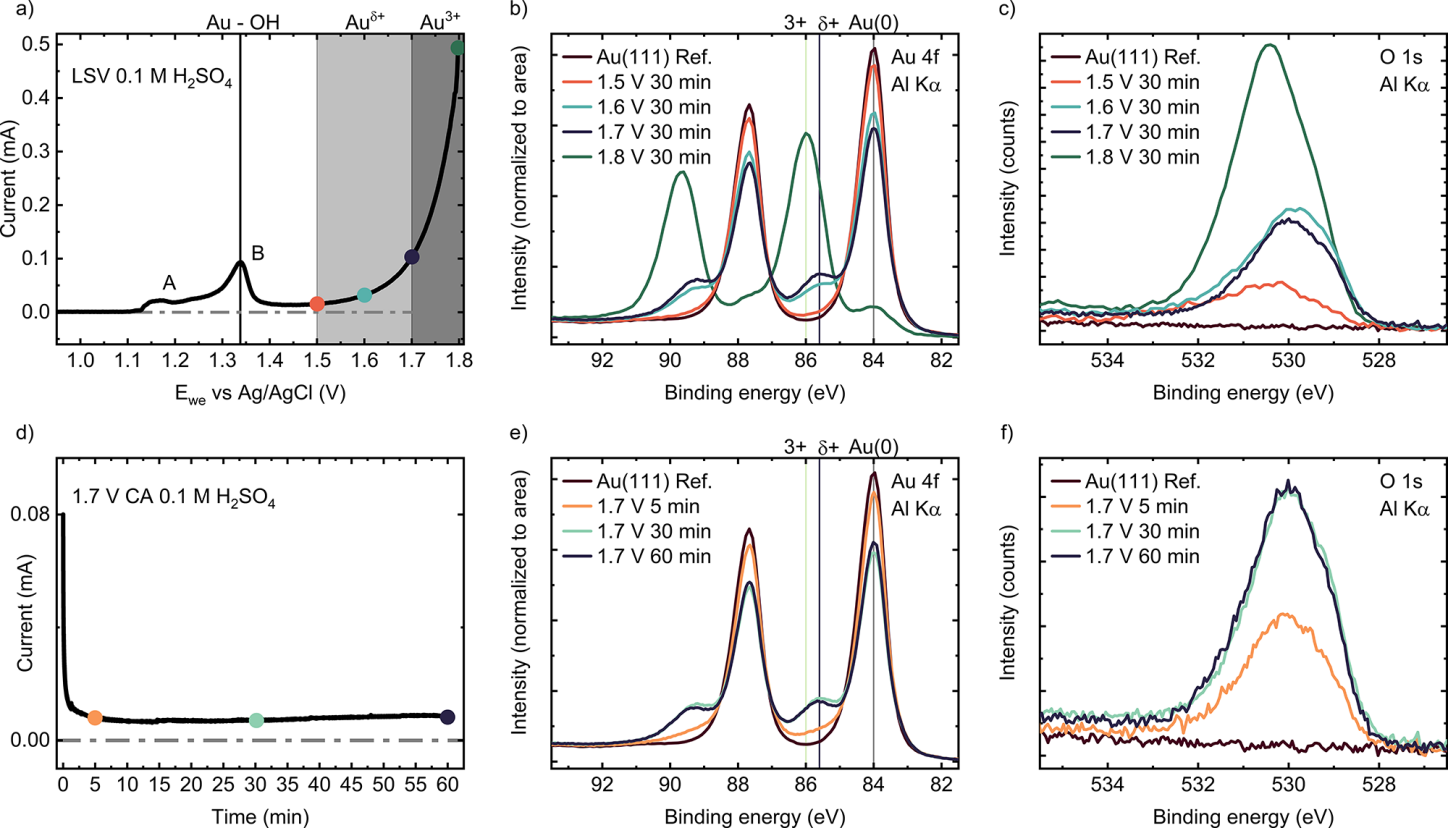
(a) Linear sweep voltammetry of Au(111) in a 0.1H_2_SO_4_ solution. A and B indicate surface oxidation waves. Orange, cyan, dark blue, and green dots mark potentials applied before acquiring data in (b,c). (b,c) Evolution of the Au 4f and O 1s lineshapes with the applied potential after 30 min CA. (d) Current recorded during a 60 min CA at 1.7 V. Orange, green, and purple dots mark the position in the CA of the corresponding XPS spectra in (e,f). (e,f) Evolution of the Au 4f and O 1s lineshapes as function of the CA duration.

The Au 4f spectrum of the clean gold electrode (brown line in Figure 1b) shows the characteristic metallic state, comprising a doublet of spin–orbit components (4f_5/2_ and 4f_7/2_) with an energy splitting of approximately Δ = 3.7 eV.^27^ The lower B.E. component, 4f_7/2_, is located at 84.0 eV.

After holding the sample potential at 1.5 V for 30 min (orange line in Figure 1b), we observe a decrease in the intensity of the metallic component, simultaneously with the emergence of a new spectral feature with a B.E. of 85.6 eV that we tentatively assign to a Au^δ+^ oxidation state. After 30 min of CA at 1.6 V (cyan) and 1.7 V (dark blue) the intensity of the Au^δ+^ component increases with respect to the previous applied potential. The spectra after the CA experiment at 1.8 V (green in Figure 1b), well within the OER regime, show a dominant Au 4f_7/2_ component at a binding energy of 86.0 eV, characteristic of the Au^3+^ oxidation state,^17,28^ and an almost complete attenuation of the metallic gold component. From the intensity decrease of the metallic Au^0^ component in Figure 1b and the inelastic mean free path and information depth of Au 4f electrons (1.6 and 4.8 nm at 1402 eV kinetic energy, respectively),^29^ we estimate that an equivalent to a 1.2 nm thick Au^δ+^ and 4.3 nm thick Au^3+^ films are formed at 1.7 and 1.8 V, respectively. Note that the thickness of the Au^δ+^ for a given potential varies across emersion experiments, Figure S4. The equivalent thickness varied from 0.7 to 1.2 nm.

Figure 1c shows the O 1s line shape measured after the CA experiments at 1.5 V (orange), 1.6 V (cyan), 1.7 V (dark blue), and 1.8 V (green). Qualitatively, two O 1s components are evidenced and correlated to the formation of the Au^δ+^ (1.5–1.7 V) and Au^3+^ oxides (1.8 V). The O 1s peak of the Au^δ+^ shifts to lower B.E. with increasing applied potential, from 530.2 eV after CA at 1.5 V to 529.8 eV after CA at 1.7 V. Conversely, the B.E. measured of the Au^3+^ O 1s is stable at 530.4 eV, with increasing intensity at larger anodic potentials. XPS analysis of residual C 1s and S 2p is shown in Figure S5. Assesing the amount of impurities in our experiment, STM imaging after 30 min of open-circuit potential (OCP) in 0.1 M H_2_SO_4_ and rinsing reveals the Au(111) herringbone reconstruction with adsorbed C impurities, Figure S6. From the XPS and STM impurities analysis we estimate an approximately 0.4–0.5 monolayer (ML) coverage of graphitic C.

OCP monitoring was performed under the same conditions as the CA experiments to assess the stability of the Au^δ+^ and Au^3+^ films after emersion. Figure S7a shows the evolution of the OCP following CA at 1.8 V for 30 min. The new OCP reached after 30 min does not correspond to a potential at the reduction wave observed in the CV, Figure S7b, indicating a preservation of the Au^3+^ state.

The progression of Au^δ+^ with time at a fixed potential was investigated by CA. Figure 1d shows the current measured over 60 min at 1.7 V. The current decreases during the first 5 min and then it reaches a plateau above 0.005 mA. Figure 1e compares the Au 4f spectra after CA for 5 (orange), 30 (cyan), and 60 (dark blue) min at 1.7 V. After 5 min, the 85.6 eV Au^δ+^ contribution is already present. Extending the CA to 30 and 60 min yields a similar intensity of the Au^δ+^. The intensity of the corresponding O 1s spectra, Figure 1f, also increases between 5 and 30 min CA and then saturates at 60 min. No binding energy shifts or additional O 1s components are identified as a function of the CA duration at 1.7 V.

Figure 2 shows the structural characterization of the Au^δ+^ film formed upon CA at 1.7 V investigated using low-temperature (LT-)­STM/nc-AFM in UHV. Large-scale image of the surface after polarization, Figure 2a, shows a rough surface with island-like oxide structures, without apparent preference of oxide growth from step edges, as it occurs during the oxidation of other metals such as Pt(111)^30^ and Cu(111).^31^ LT-STM/nc-AFM images and low-energy electron diffraction (LEED) reference of clean Au(111) are provided in Figure S8. Stable STM imaging of the oxidized film required elevated sample biases (>2 V), indicating its reduced electronic conductance. On the oxidized regions, Figure 2b, no long-range order is observed, with an apparent corrugation of the island of about 150 pm. Within these islands, STM resolves seven-membered, ring-like motifs, Figure 2c. nc-AFM performed on the oxide-free areas, Figure 2d, shows the 1 × 1 Au(111) atomic structure with a 2.9 Å ± 0.1 Å lattice constant and no periodic herringbone reconstruction.

**Figure 2:**
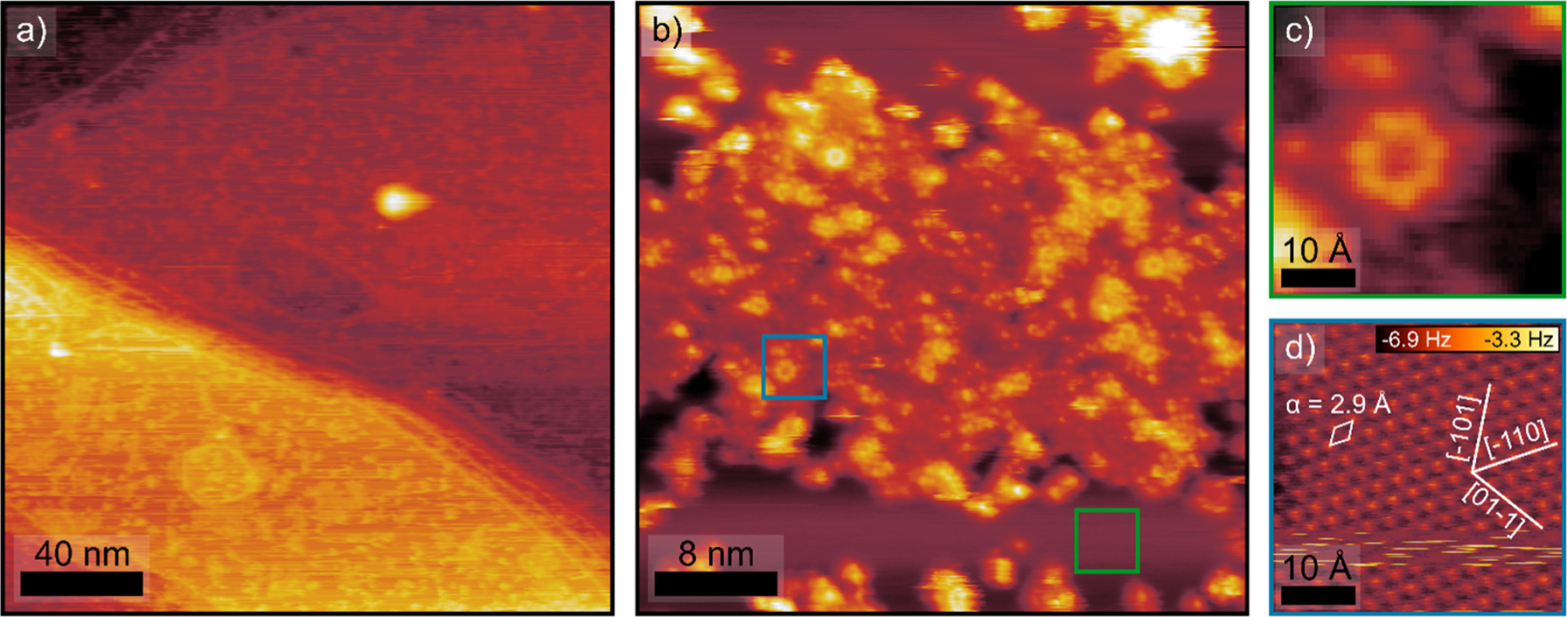
(a) STM overview of the Au(111) surface after 30 min CA at 1.7 V in 0.1 M H_2_SO_4_. *V*_sample_ = 2.5 V, *I*_set point_ = 5 pA. (b) Detail of a Au^δ+^ island. Green and blue squares mark the areas of images (c,d). *V*_sample_ = 2 V, *I*_set point_ = 5 pA. (c) Magnification of one of the 7-ring structures identified in the Au^δ+^ islands. (d) nc-AFM image of 1 × 1 Au(111) without the herringbone reconstruction *V*_sample_ = 50 mV, amplitude = 150 pm.

Double-line structures with a 2.5 nm separation running along some of the oxide islands can be identified on regions of the sample, as shown in Figure S9. For thicker Au^δ+^ films, STM imaging was not feasible due to a more insulating character. nc-AFM resolved a surface roughness arising from small (2–4 nm wide) Au^δ+^ clusters lacking internal crystalline order, Figure S10a,b. The local structural disorder inferred from nc-AFM is further supported by the absence of sharp LEED spots, particularly at low electron energies, as shown in Figure S10c.

We followed the surface evolution in UHV after 30 min of CA at 1.7 V, Figure 3. The Au 4f_7/2_ spectrum immediately after emersion shows a component at 85.6 eV that decreases steadily with time, while the metallic line at 84.0 V increases, Figure 3a. Consistently, the contribution of the O 1s at about 529.8 eV diminishes over the same interval, Figure 3b. The evolution of the photoelectron peak heights related to Au^0^ (obtained from the 4f_7/2_ spectra) and Au^δ+^ (obtained from the O 1s spectra) with time are shown in Figure S11. Au^0^ increases progressively, while Au^δ+^ decreases accordingly. Assuming a linear decay of the Au^δ+^ signal, a decay rate of 1.6%/h is obtained. Thus, after ca. 61 h in UHV (1 × 10^–9^ mbar), 95% of the surface Au^δ+^ has been reduced to Au^0^. Figure S11 also confirms that Au^δ+^ follows the same decay tendency in the absence of X-ray beam exposure, implying that the surface oxide reduction is not a beam-induced effect.

**Figure 3:**
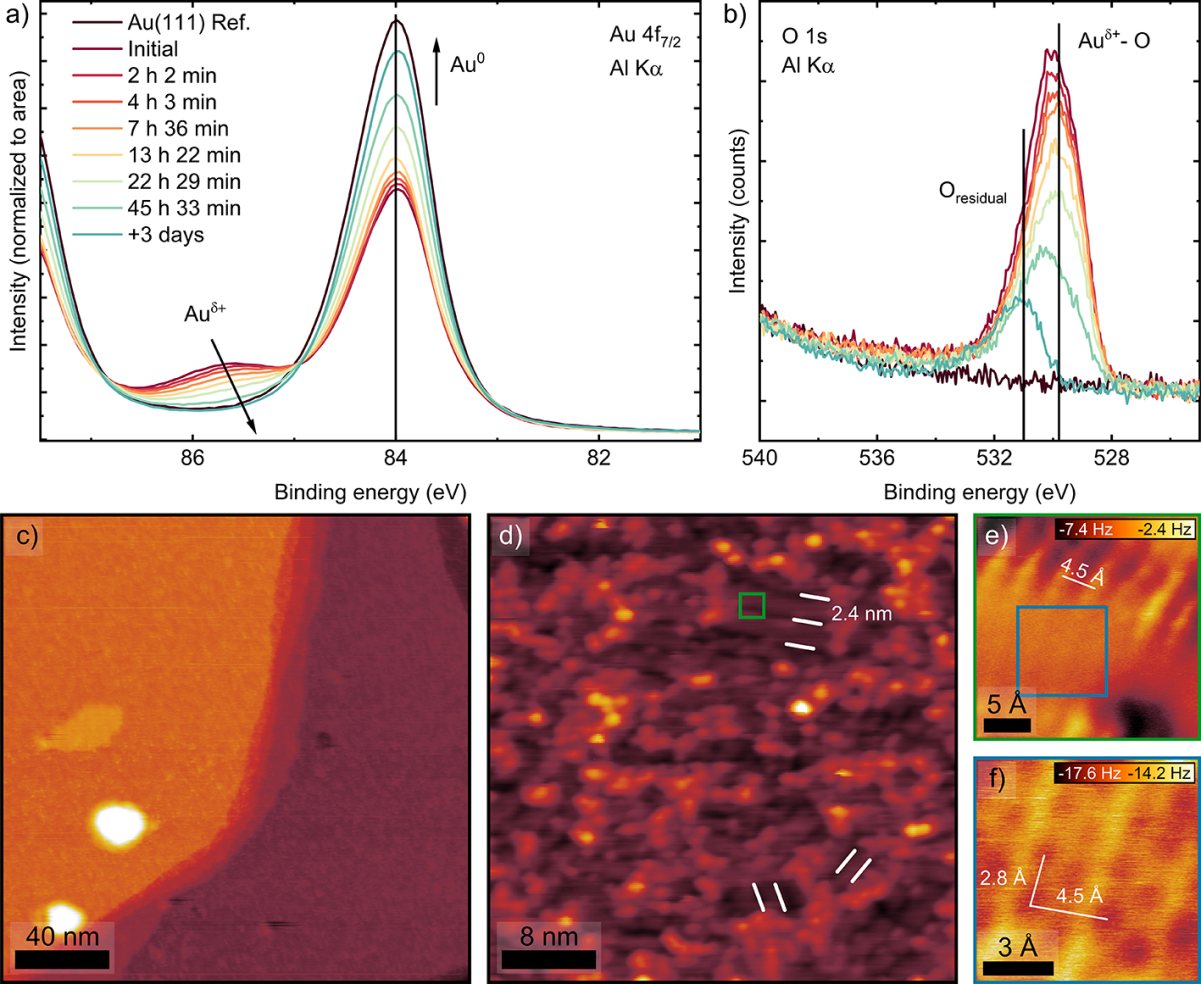
Spontaneous reduction of Au^δ+^ in UHV. (a) Au 4f_7/2_ and (b) O 1s XPS spectra after the spontaneous reduction of Au^δ+^ in UHV during 72 h. (c) Large-scale STM (4 K) overview of the Au^δ+^ film in Figure 2 (1.7 V, 30 min) after reduction in UHV for 72 h. No oxide islands are visible at this stage. *V*_sample_ = 1 V, *I*_set point_ = 20 pA. (d) STM detail of (c) showing line structures in three orientations rotated 120° with respect to each other. The apparent separation between the lines is 2.4 nm. *V*_sample_ = 1 V, *I*_set point_ = 20 pA. The green square marks the region imaged in (e). (e) nc-AFM detail of the boxed region in (d), revealing a continuous layer of height-modulated atomic rows oriented perpendicular to the long axis of the line motif. *V*_sample_ = 5 mV, amplitude = 120 pm. (f) Atomic-scale nc-AFM image of rows in (e), showing a lattice parameter of 2.8 Å. *V*_sample_ = 2 mV, amplitude = 120 pm.

The structural analysis of the Au^δ+^ oxide spontaneously reduced in UHV is shown in Figure 3c–f. Figure 3c displays a large-area STM image of the surface after 72 h in UHV. Upon reduction, stable STM at 1 V becomes possible, reflecting the enhanced conductance relative to the oxidized state. The surface is now homogeneously covered by parallel line motifs arranged in three equivalent domains rotated by 120°. The apparent separation between adjacent lines is 2.4 ± 0.1 nm. Close up view constant-height nc-AFM imaging of these structures, Figure 3e, shows a height-modulated atomic corrugation in the form of rows perpendicular to the STM lines. The separation between rows is 4.5 Å ± 0.1 Å. Note that a darker/lighter contrast in constant-height mode translates into smaller/larger tip-sample distances. Within these rows, we observe a rectangular unit cell of 2.8 × 4.5 Å ± 0.1 Å, Figure 3f.

In addition to UHV, we explore the reduction of Au oxide in different atmospheres. In situ XPS under 10 mbar H_2_O backfilling yields a much faster reduction of the Au^3+^ oxide, obtained by 30 min CA at 2 V, than in UHV, Figure S12. Additionally, we evaluated the air stability of the Au^δ+^ oxide film. The Au^δ+^ oxide, obtained by 30 min CA at 1.7 V, was exposed to air at controlled pressures (0.1 mbar–1 bar) without X-ray illumination and then measured in UHV, Figure S13. The Au^δ+^ component decreases stepwise with each exposure, accumulating an ∼33% loss over ∼3 h of air exposure and UHV measurement.

To further assess the intrinsic stability of the Au^δ+^ phase, we conducted in situ XPS measurements during laser heating under UHV (1 × 10^–9^ mbar), from room temperature (RT) to 200 °C. Cyclic monitoring of the Au 4f and O 1s components was carried out, increasing the temperature in 50 °C steps, and the sample held at each set point during acquisition. The laser heating takes one min for each temperature increment. Figure 4a shows the in situ evolution of the Au 4f line shape upon heating the Au^δ+^ film, produced by 30 min CA at 1.7 V, from room temperature to 200 °C. The Au^δ+^ Au 4f_7/2_ component decreases with temperature while the intensity of the metallic Au^0^ peak grows concomitant with a decrease in the O 1s intensity under the same conditions, Figure 4b. At 200 °C, ∼10% of the Au^δ+^ component remains, reducing during cool-down (∼40 min).

**Figure 4:**
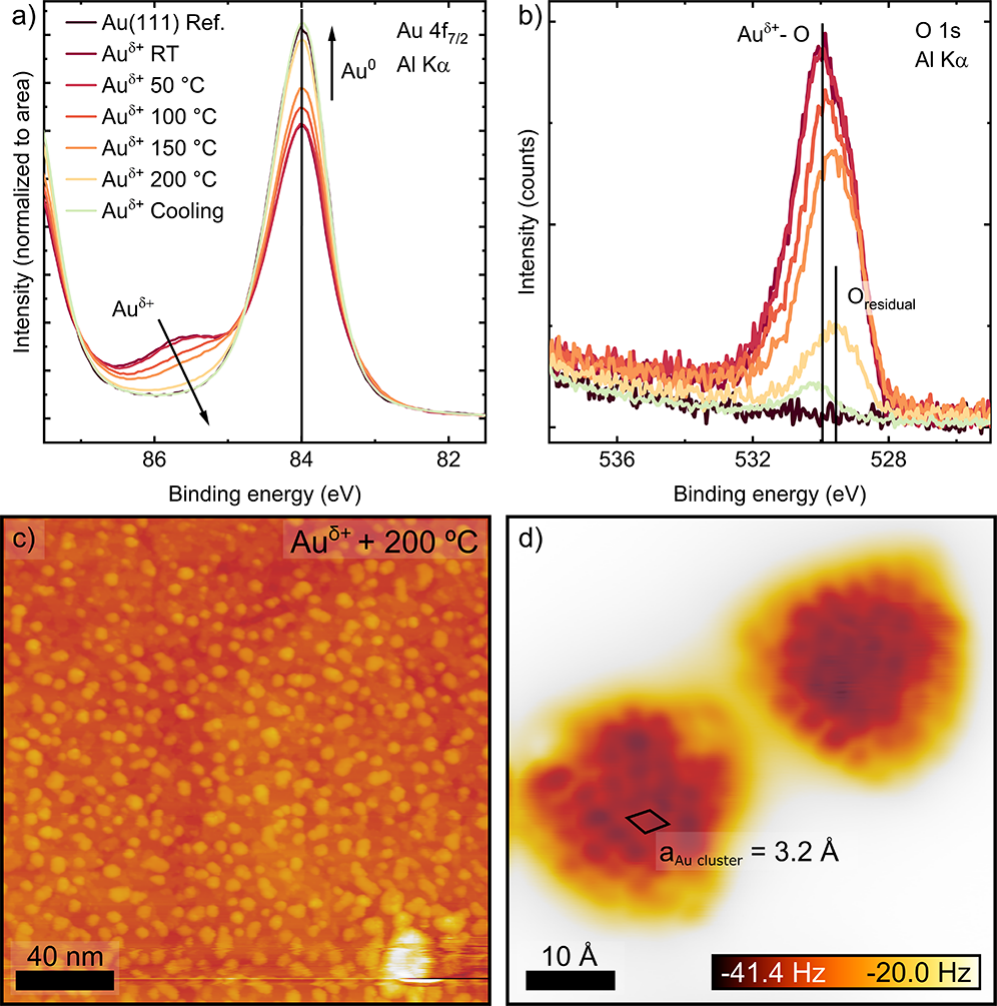
Thermal reduction of Au^δ+^ in UHV. (a) Au 4f_7/2_ and (b) O 1s spectra of the thermal reduction of Au^δ+^ (1.7 min, 30 min) in UHV from RT to 200 °C. (c) Large-scale STM image of the Au^δ+^ surface after 200 °C thermal annealing. The surface is now decorated by cluster structures. *V*_sample_ = 2.25 V, *I*_set point_ = 20 pA. (d) Close-up view nc-AFM image of representative clusters observed in (b), unveiling internal atomic structure. The apparent lattice parameter of the clusters is 3.2 Å (black rhombus). *V*_sample_ = 4 mV, amplitude = 150 pm.

We conducted an analogous structural study by LT-STM/nc-AFM to complement the chemical analysis of thermal reduction by XPS. The sample was annealed on the UHV manipulator in 50 °C increments and, after each step, transferred into the microscope for imaging at *T* < 4.8 K. The heating takes 5 min for each temperature step. The surface morphology remained unchanged until the 200 °C annealing. Figure 4c presents a large-area STM image of the thermally reduced Au^δ+^ oxide, produced by 30 min of CA at 1.7 V, after 5 min of annealing at 200 °C in UHV. The surface appears decorated by flat clusters with an apparent 2–3 nm length by STM. High-resolution nc-AFM imaging resolves a hexagonal crystalline in-plane periodicity within the clusters, with a unit cell of 3.2 Å ± 0.1 Å (Figure 4d). The corresponding LEED pattern of this surface, Figure S14, displays two hexagonal spot patterns with the same orientation: a less defined inner one and a sharp outer pattern. We attribute the inner spots to the cluster periodicity 3.2 Å and the outer ring to the Au(111) substrate periodicity, 2.9 Å. Note that shorter reciprocal space distances translate into larger periodicities and smaller periodic domains (clusters) in real space produce more diffuse LEED spots.

## Discussion and Conclusion

Our quasi-in situ XPS measurements provide solid evidence for the formation of an intermediate electrooxidized state of gold, Au^δ+^ (0 < δ < 3), precluding the Au^3+^ electrooxidized state. The Au^δ+^ oxide is obtained by the polarization of Au(111) in 0.1 M H_2_SO_4_ at potentials that are lower than required for Au^3+^ oxidation. XPS resolves distinct Au 4f and O 1s features that increase with increasing potential (from 1.5 to 1.7 V) and polarization times (from 5 to 30 min) until saturation (60 min). Hence, Au^δ+^ denotes a surface-limited, oxidized Au phase identified by a positive Au 4f shift relative to Au^0^ and the absence of Au^3+^ features under our conditions. The notation is descriptive and does not imply a uniform integer oxidation number per site. Reduction of the Au^δ+^ oxide back to metallic Au^0^, proceeds spontaneously in UHV and results in a height-modulated periodic superstructure on Au(111) without recovering the initial herringbone reconstruction.

The Au^δ+^ thickness at a given potential varies across emersion experiments, Figure S4, yielding specimens with different coverages and, consequently, distinct structural evolutions under the same polarization conditions. At lower coverage, STM/nc-AFM after 30 min of CA at 1.7 V, Figure 2, shows oxide islands without step-edge preference. The incomplete Au^δ+^ film is amorphous, with exposed 1 × 1 Au(111), consistent with the lower thickness estimation of ∼0.7 nm from the Au 4f attenuation. High-resolution STM reveals atomic-scale protrusions and occasional seven-member ring-like motifs, i.e., local patterns compatible with short-range Au–O connectivity, but no long-range order. At higher coverage, a compact, cluster-corrugated film is formed, Figure S10. While XPS after 30 min of CA at 1.7 V places the surface in the Au^δ+^ regime with no resolvable Au^3+^, nc-AFM measurements do not allow us to distinguish between amorphous Au^δ+^ and Au^3+^ structures without local order. Notably, the coverage-dependent morphology, together with the joint Au 4f/O 1s analysis, supports a spectroscopic separation between the Au^δ+^ and Au^3+^ regimes under our conditions.

As the CA polarization potential for Au(111) samples increases, the O 1s spectra shift from ∼530.2 eV (polarization at 1.5 V) to ∼529.8 eV (polarization at 1.7 V) and the intensity then grows until saturation after 30 min CA at 1.7 V, Figure 1c,f. Au^3+^-related O 1s XPS signal sets at a higher B.E., near ∼530.4 eV. Considering the range of possible Au (oxy)­(hydro)­oxide species that can be contributing to the O 1s spectrum, and the proximity of the reported binding energies for some of those species (529.3, 529.4, and 530.3 eV for plasma generated Au–O,^32,33^ 529.7 eV for a surface Au–O,^15^ 529.1 and 530.0 eV for the two O sites in Au_2_O_3_ ^32^, 530 and 531 eV for –OH,^16,28^ 530.8 eV for –OOH and 531.6 for Au­(OH)_3_·H_2_O,^17^ and 531.8 eV for H_2_O adsorbed on Au^16^), we analyze the O 1s spectra here only by comparing their intensity rather than assigning specific Au–O/OH configurations. A proper deconvolution to accurately identify the surface species contributing to the 1s-O spectrum would greatly benefit from theoretical calculations and the use of additional experimental techniques to support the interpretation. This would also help define the chemical environment and oxidation state of Au^δ+^. In line with this, recent operando HERFD–XANES studies inferred a short-lived, surface-confined Au^1+^-like intermediate under anodic conditions, compatible with our observation of an Au^δ+^ phase.^14^

In parallel, varying the polarization time at 1.7 V shows Au^δ+^ emerging within 5 min and then saturating at 30 min, Figure 1e,f. The polarization current decays to a nonzero plateau, Figure 1d. Based on our results, thermodynamic drive (higher anodic potential) and kinetic accessibility (time under polarization) act together to evolve the Au^δ+^ oxide detectable. While oxidation likely initiates at lower potentials, a stabilized, surface-limited Au^δ+^ requires both increased bias and finite dwell.

This observation is consistent with Conway’s early model for the electrooxidation of Au(111) in acidic media, which described the initial formation of a two-dimensional (2D) oxide layer.^18^ This 2D oxide was theorized to be created by a place-exchange mechanism involving adsorbed oxygen species and gold atoms from the topmost surface layers of the substrate. According to Conway et al., the growth of this 2D oxide is kinetically self-limited by the mobility of Au atoms from the surface and deeper layers, and only at sufficiently high fields (potentials) is ion migration induced deep enough into the bulk to produce a bulk oxide. The kinetic nature of this place exchange explains the necessity for extended CA times to fully evolve the Au^δ+^ phase in our experiments. In parallel, the observed gradual formation of the transient Au^δ+^ state with the increasing applied potentials agrees with the presence of a current plateau in the characteristic CV preceding the OER regime and suggests a gradual oxide growth.^1,11,13,18^

Previous XPS studies largely described the mechanism based on a dual Au^0^/Au^3+^ framework,^16,17,28^ treating possible Au^δ+^ signatures as part of the evolving Au^3+^ spectra rather than as a discrete component. In this context, Au^δ+^/Au^1+^-like contributions have been reported or indicated in prior XPS-based studies, but they were generally not treated as an independent surface phase. Here, we go beyond these observations by directly resolving the Au^δ+^ state chemically and structurally and demonstrating its surface-limited transient nature. UHV analysis provides a very low oxygen chemical potential that promotes reduction, as demonstrated in our experiments, where the Au^δ+^ film after 30 min CA at 1.7 V is reduced over 72 h to Au^0^ under UHV, Figure 3. Controlled exposure to air (from 0.1 mbar to 1 bar), followed by UHV analysis, reveals a faster reduction (about 1/3 over 3 h) rate than the spontaneous decay observed in UHV. Our results also show a significant reduction effect when measuring XPS under water vapor backfilling, Figure S12, which could also challenge the interpretation of the results under more realistic environments.^16^ Similar observations have been reported for the apparent reduction of copper oxides during NAP-XPS experiments, attributed to beam-induced water hydrolysis.^34^ In parallel, it is important to note that higher relative humidity is known to introduce adventitious carbon,^35^ which, upon adsorption, can reduce the energy barrier for water splitting and modify surface chemistry,^36^ effectively leading to Au oxide reduction. Based on our results, short polarization times do not lead to sufficient accumulation or stability of the Au^δ+^ intermediate, challenging its detection under UHV conditions. While neither factor is critical in the Au-oxidation to Au^δ+^ in the present study, they underscore the value of transparent protocols and benchmarking, systematically eliminating and documenting potential uncertainties (transfer chronology and polarization times; UHV evolution; reporting of C 1s/O 1s/anion signatures; verification of residual electrolyte or air-derived adsorbates). Such practices would support a sequential, gradual understanding of the more complex oxidation mechanisms.

Recent electrochemical studies have identified the presence of an electrochemically produced Au^1+^ oxide, distinct from the Au^3+^, in alkaline media through in situ near-ambient pressure (NAP)-XPS^15^ measurements of polycrystalline gold. Note that in alkaline solutions, the abundant hydroxide ion (–OH) facilitates the formation of Au–OH species, leading to different reaction pathways. Relevantly, analogous observations of a Pt^δ+^ state have been made for platinum during in situ electrochemical XPS studies in alkaline media.^37^ Here, our current findings on Au^δ+^ in acidic conditions further corroborate the idea of transient, oxidation states playing a crucial role in the initial stages of noble metal oxidation. This suggests a common underlying mechanism in the early stages of noble metal oxidation, involving the formation of a thin film before bulk oxidation, with a transition through an M^δ+^–O state.

The spontaneous reduction of the Au^δ+^ oxide islands leads to a surface uniformly covered in line structures with 2.5 nm spacing, Figure 3d, and three rotational domains reminiscent of the herringbone reconstruction of Au(111), Figure S8. However, we do not observe the reconstructed Au surface, Figure S8b, but rather a height-modulated overlayer with a 2.8 Å × 4.5 Å ± 0.1 Å unit cell strikingly reminiscent of a Moiré pattern, Figure 3e,f. On the other hand, the thermal reduction produces a surface covered in crystalline clusters with a unit cell, 3.2 Å ± 0.1 Å, ∼12–13% larger than the Au(111) nearest-neighbor spacing, Figure 4d. To the best of our knowledge, metallic Au clusters with such a structure have not been reported before. Both end-states of Au^δ+^ oxide include an O 1s fingerprint, although at different B.E.; 531.1 and 529.6 eV for the spontaneous and thermally reduced surface, respectively, Figures 3b and 4b. The level of C–C impurities does not increase during reduction in UHV, whereas the C signal disappears upon 200 °C annealing, Figure S5c. Thus, we interpret the structures found after Au^δ+^ oxide reduction as ultrathin Au oxide phases. The lack of spectroscopic evidence of the Au-oxidation state in these films in the Au 4f spectra is comparable to the case of ultrathin 2D copper oxides. The identification of changes in Cu-oxidation state at the surface is challenging using lab-source XPS, and requires analyzing minute changes in the Cu LMM and valence band spectra by synchrotron radiation XPS and comparing the results with a reference compound.^38^

The binding energy shift of the Au^δ+^ oxide, positioned between metallic gold and bulk Au^3+^ oxide, could be influenced by size-dependent effects inherent to small oxide clusters. While often discussed in the context of metallic nanoparticles, similar quantum confinement or altered local coordination environments within nanoscale oxide clusters can modify their electronic structure and, consequently, their photoemission binding energies. Such effects could lead to a less pronounced positive shift than expected for bulk Au^3+^ oxide, effectively contributing to the “δ” oxidation state. This has implications for the oxidation pathways and reactivity of surfaces subjected to redox cycling before the onset of a reaction. Such a situation is exemplified in the oxidation of Au by using reactive O sources. Whereas plasma oxidation of polycrystalline Au results in a δ+ state,^39^ cluster oxidation is reported to occur from a direct metallic Au to a Au_2_O_3_ phase (Au^3+^).^40^

In conclusion, our comprehensive chemical and structural analysis of the Au electrode evolution under anodic oxidation evidences a surface-limited Au^δ+^ (0 < δ < 3) oxide layer that forms and grows to a saturation thickness before any bulk-like Au^3+^ oxide is detected. The identification of a discrete intermediate state significantly advances the understanding of the gold electro-oxidation mechanism, moving beyond the broadly assumed one-step (Au^0^/Au^3+^) model to unveil the crucial role of a transient, surface-limited species. Whether an electrochemical reduction of Au^3+^ proceeds through an Au^δ+^ intermediate state before reaching Au^0^ represents an interesting avenue for future studies. This intermediate state is likely a consequence of a substoichiometric Au/O ratio, potentially combined with confinement-related electronic effects. While Au^δ+^/Au^1+^-like spectral features have been reported in earlier studies, our combined quasi-in situ XPS and atomic-scale microscopy establishes this state as a distinct, surface-limited intermediate with well-defined formation and decay behavior. Our results thus provide direct quasi-in situ chemical and structural evidence for distinct stages of gold oxidation, supporting a two-step framework: first, nucleation and self-limiting growth of a surface Au^δ+^ oxide; second, at higher potential/charge, the development of a thicker, bulk-like Au^3+^ oxide.

These findings illustrate the feasibility for a generalized “multistep” oxidation mechanism across noble metals and suggest a common characteristic where the active OER phase is not necessarily only the highest oxidation state of the metal but rather a surface-confined and potentially substoichiometric species. This work demonstrates the relevance of in situ and quasi-in situ experimental approaches with full environmental control to explore the chemical and structural evolution of catalysts due to operando conditions, providing a more comprehensive picture of their dynamic behavior.

We provide direct experimental evidence for a transient, metastable Au^δ+^ oxide formed during Au electrooxidation, together with a protocol that links electrochemical treatment to ex situ UHV characterization and reveals route-dependent structural and spectroscopic outcomes. While our data robustly establish the existence and transient nature of this Au^δ+^ state, we recognize that a definitive atomic-scale assignment of the Au^δ+^ structure will be best supported by a follow-up combined experiment–theory study. The present work provides a set of well-defined experimental constraints—spectroscopic fingerprints, coverage limits, stability trends, and morphology evolution—that can serve as benchmark targets for emerging constant-potential and explicitly solvated models^41^, and for approaches capable of treating metastable, nonstoichiometric, and nonperiodic surface motifs.^42^ We anticipate that this experiment–theory synergy will sharpen mechanistic understanding of Au-oxidation beyond simplified pictures and enable more predictive descriptions of electrocatalytic surface transformations.^43,44^

## Methods

### XPS Analysis

The Au(111) single crystals were prepared in UHV by cycles of Ar^+^ sputtering and annealing until a clear herringbone structure was observed by LEED and no impurities were present in XPS. XPS experiments were performed using a Phoibos 150 NAP analyzer and a μFOCUS 600 X-ray monochromator (Specs GmbH), Al Kα anode (1486.4 eV, spot size 0.3 mm) in the CERIC facilities at Charles University and at the University of Basque Country. The core-level spectra were recorded with a pass energy of 20 eV, step size of 0.05 and 0.1 eV, and dwell time of 100 ms. XPS data were analyzed and fitted using KolXPD^45^ and CasaXPS. The base pressure in both systems was below 1 × 10^–9^ mbar. The metallic gold peak, Au 4f_7/2_ = 84.0 eV, was used for the energy calibration of the spectra.^27^ Quantitative cleanliness tracking (C 1s, S 2p), Figure S5, shows no detectable C–O or S–O related contributions. XPS Analysis of the adventitious carbon level in our experiments versus 1 ML of DBBA molecules on Au(111)^46^ is shown in Figure S5c.

### STM/nc-AFM and LEED Characterization

Simultaneous STM/nc-AFM images were acquired in an Omicron POLAR-SPM at 4.8 K using qPlus sensors^47^ (resonance frequency of ca. 28 kHz, *Q* factor of ca. 10,000) with an electrochemically etched W tip at the Department of Surface and Plasma Science at the Charles University, and Unisoku USM1800 at 4 K using qPlus sensors (resonance frequency of ca. 29 kHz, *Q* factor of ca. 14,000) with a Pt/Ir tip at the University of the Basque Country. The tips were prepared on Cu(111) with partial oxide coverage to obtain a CuO_*x*_ tip termination^48^ until a change in the resonance frequency <−1.5 Hz at 0.1 V bias and 100 pA was obtained in tunneling contact, and the contact potential difference between tip and sample was <0.2 V.^49,50^ LEED patterns (LEED 800 OCI) were taken at the Department of Surface and Plasma Science at the Charles University.

### Electrochemical Test

Measurements of the EC response were acquired with a potentiostat/galvanostat PGSTAT 204 from Metrohm-Autolab and Nova software. The electrochemical measurements were carried out in an electrochemical cell (EC cell), which was connected to the UHV chamber via a load lock.^21,22^ The EC measurements were carried out in a three-electrode configuration. UHV-prepared Au(111) was used as the working electrode, a leakage-free miniature Ag/AgCl (Edaq ET072) as the reference electrode, and an Au wire served as a counter electrode. The H_2_SO_4_ electrolyte solutions were prepared with Suprapur (Merck) H_2_SO_4_ (96%) and MiliQ water with resistivity of 18.2 MΩ·cm at 25 °C (Merck Millipore Milli-Q system). The electrolyte was purged with Ar (6 N, 99.9999% purity) for at least 1 h prior to measurements to remove the dissolved oxygen content. Further details of the EC setup, cleaning procedure, and emergence experiments can be found in the Supporting Information, together with a schematic of the EC-UHV setup, Figure S1.

## Supplementary Material



## Data Availability

The data of the experiments for this manuscript have been made available at Zenodo: DOI: 10.5281/zenodo.16615611.

## Notes

Description of the XPS and SPM equipment; description of the EC-UHV setup, the cell cleaning and emersion experiment; estimation of the OER onset; details of the CV sample conditioning; comparison of the Au 4f signal between emersion experiments; estimation of the amount of impurities across experiments; STM characterization after OCP and sample rinsing; OCP evolution of Au oxide; clean Au(111) STM/nc-AFM/LEED reference; detail of clean Au on Au oxide; thick Au^δ+^ oxide STM/nc-AFM/LEED reference; estimation of the Au^δ+^ reduction rate in UHV; comparison of Au^3+^ reduction in UHV and water; effect of air exposure on Au^δ+^; and LEED detail of Au^δ+^ oxide after thermal reduction (PDF)
