# Supplementary material for: Direct Experimental Evidence of Transient Auδ+ Oxide in Au Electrooxidation
Source: J Am Chem Soc. 2026 Feb 25;148(12):12587–96. doi: 10.1021/jacs.5c13087 (PMC13047530; doi:10.1021/jacs.5c13087)
Supplement: Supplementary file 1 [file ja5c13087_si_001.pdf]

Direct Experimental Evidence of Transient $Au^{\delta+}$ Oxide in Au Electrooxidation

Jesús Redondo[1,2,3,ɫ,*], *Ane Etxebarria*[3,ɫ], *Pankaj Kumar Samal*[1,ɫ], *Llorenç Albons*[1], *Roser Fernandez Climent*[1], *Sabine Auras*[2], *Břetislav Šmíd*[1], *Xiaohui Ju*[1,4], *Peter Matvja*[1], *Frederik Schiller*[2], *Martin Setvín*[1], *Josef Mysliveček*[1,*], *Sara Barja*[2,3,5,6,*]

[1] Department of Surface and Plasma Science, Charles University, Prague, Czech Republic

[2] Centro de Física de Materiales CFM-MPC (CSIC-UPV/EHU), San Sebastián, Spain

[3] Departamento de Polímeros y Materiales Avanzados: Física, Química y Tecnología, University of the Basque Country, San Sebastián, Spain

[4] Department of Chemistry and Biochemistry, Mendel University in Brno, Brno, Czech Republic

[5] Donostia International Physics Center, San Sebastián, Spain

[6] Ikerbasque, Basque Foundation for Science, Bilbao, Spain

[ɫ] J.R., A.E. and P.K.S. contributed equally to this work

*Email: jesus.redondo@mff.cuni.cz, josef.myslivecek@mff.cuni.cz, sara.barja@ehu.eus

Keywords: electrochemistry, electrooxidation, XPS, STM, nc-AFM

The EC setup

The EC setup involved in this work and in the previous studies[1,2] consists of 5 subsystems, Figure S1: (A) the EC cell, (B) the bottles and tubing connected to the EC cell, (C) the argon distribution panel for degassing and propelling liquids and maintaining an inert atmosphere during sample transfer (D) the bubblers for pressure equalization and flow control, and (E) UHV load-lock chamber for the sample transfer under inert atmosphere.

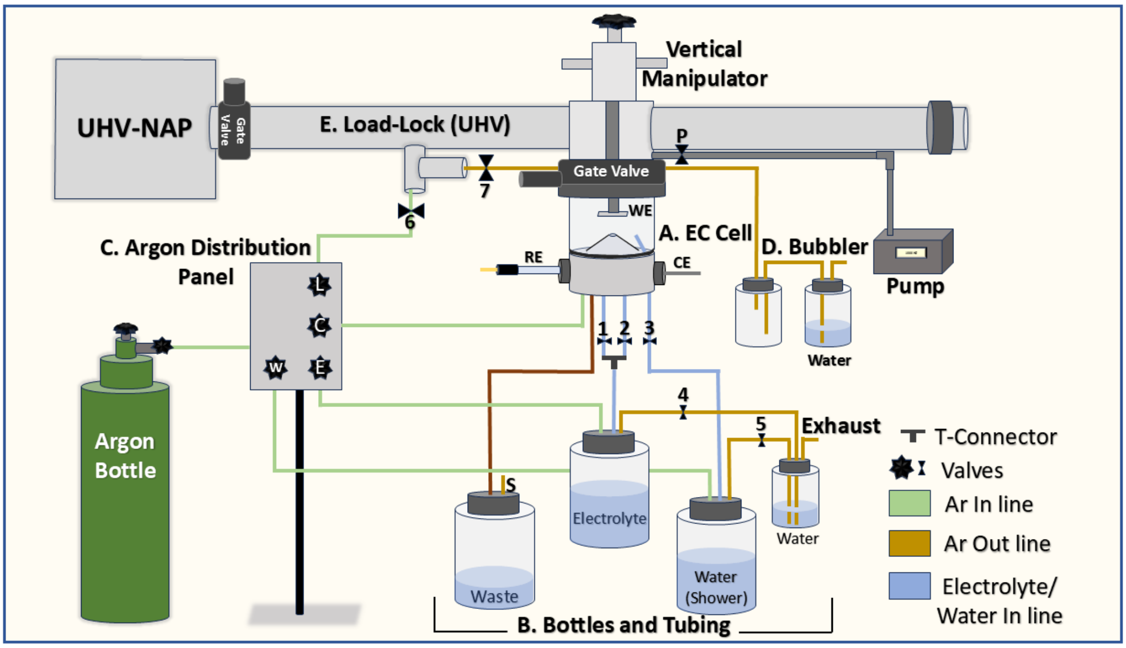

Supplementary figure 1. The EC-UHV setup.

The EC cell is intended for measurements in a stationary electrolyte. The cell is fabricated from PCTFA (Kel-F), has two separate electrolyte inlets including compartments for the reference electrode (RE) and for the counter electrode (CE)[3], inlets for Ar gas, and for ultrapure water for sample rinsing ("shower"), and outlet for water/electrolyte drain ("waste")[4]. Tubing and fittings for gas and liquid distribution are from chemically resistant plastic (PTFE, PFA, ETFE). The electrolyte bottle is made of PFA, other bottles are borosilicate glass. Argon 6.0 (Linde) is distributed from a 50 L cylinder using a high-purity gas pressure regulator Linde Red Line C200hv/2A and stainless-steel tubing to Ar distribution panel (C). Before opening the bottle, the Ar steel tubing is always evacuated using a turbomolecular pump. The bubbler (D) is arranged in a way to interrupt the backflow of the insulating water in case of under-pressure. The load-lock is constructed from standard vacuum components and allows a mechanical transfer of the samples between UHV chamber and the EC cell, and acts as an

airlock allowing controlled flooding and evacuation with 1 bar Ar. Evacuation of the load-lock is always performed using a turbomolecular pump, preventing contamination of the samples from primary pump vacuum.

Cleaning of the cell and other parts

To prevent any residual contamination and its effects on EC measurements, the cleaning procedure of the EC cell setup is as follows: the PCTFE EC cell is cleaned by soaking in a solution of sulfuric acid (Merck, 95-97%) and NoChromix (Sigma Aldrich) overnight. The next day, the cell is removed from the bath and rinsed three times with ultrapure water (MilliQ synergy UV, 18.2 MΩ cm at 25 °C), followed by boiling in ultrapure water. Rinsing and boiling procedures are repeated two more times followed by a final rinse in cold ultrapure water. The electrolyte bottle, a bottle containing water for rinsing the sample (shower bottle), and tube fittings and connectors are first cleaned using a fresh Piranha solution (a mixture of sulfuric acid (Merck, 95-97%) and hydrogen peroxide (Merck, 30%) in a 3:1 ratio), and, subsequently, using a repeated rinsing in cold ultrapure water alternated with boiling in ultrapure water. The tubing is cleaned in a separate procedure. Before installation, tube ends are kept in the sulfuric acid bath overnight and subsequently rinsed with ultrapure water. After assembly and installation of the whole EC setup, and its attachment to the load-lock, ultrapure water is passed through the tubing and the EC cell to clean the inside, followed by passing, alternately, hot and cold ultrapure water two more times followed by a final rinse in the cold ultrapure water.

Before each EC experiment, the EC cell and tubing were rinsed with ultrapure water by passing it through the tubes and the cell. Argon gas (6 N purity) was used to pressurise the bottle to push the water into the cell via valves 1 and 2, the water is then drained into the waste bottle via the waste outlet, Figure S1, brown line. In the next step, Ar gas is purged into the EC cell by opening valve C to ensure an inert atmosphere inside the cell, and drained to a bottle of ultrapure water in the place of the electrolyte bottle. This purging with Ar takes at least 60 minutes. Simultaneously, purging of the shower bottle is also performed via valve 5 while keeping valve 3 closed. Upon purging, the cell is again washed with the Ar-purged water to ensure that no oxygen remains in the electrolyte channels of the cell. After cell washing and purging with Ar, a bottle containing freshly prepared electrolyte is installed. Valves 1 and 2 are kept closed while installing the electrolyte bottle to prevent air draining in the system. The electrolyte is purged for about 30 minutes via valve 4, i.e. through the exhaust bottle, to remove

the air above the electrolyte after bottle exchange. Afterwards, the deaerated electrolyte is passed through the cell for another 30 minutes using valves 1 and 2 and keeping valve 4 closed. Valves 1 and 2 are purged separately (1 closed and 2 open and vice versa) to ensure a complete removal of air from the tubing and the cell. Simultaneously, the shower bottle purging is switched from via valve 5 to via 3. This removes any residual air from the shower line. For purging, the tubes supplying Ar are kept dipped into the liquid, while the tubes connecting to the cell are kept above the liquid level.

The emersion experiment

After cleaning and purging of the EC setup, the UHV-prepared sample is transferred from the main UHV chamber to the sample holder on the vertical manipulator in the load-lock chamber in UHV. Then Ar gas is inserted into the load-lock chamber after closing the valves to UHV and to the turbomolecular pump (P). Since all Ar supply lines emerge from one gas distribution panel, to avoid any pressure imbalance in the system, the valves C, E, and W are closed before pressurizing the load-lock. To prevent any possible insertion of air to the EC setup through the waste bottle outlet, the Ar outlet line S is kept closed using a cap. Valves C, E, W, and the cap of the outlet S are opened once the load-lock chamber is filled with Ar and valve 6 is closed. To ensure that there is no overpressure in the load-lock, the pressure is equalized to the atmospheric pressure by releasing the extra gas through the bubbler using valve 7. Immediately after pressure equalization, valve 7 is closed, and the gate valve between the cell and the load-lock chamber is opened. Then, a droplet of the electrolyte is created at the apex of the cell by slowly filling the electrolyte through the reference electrode compartment and the counter electrode compartment. The sample is finally brought into contact with the electrolyte to perform the electrochemical measurement. After finishing the measurement, the sample is detached from the electrolyte under potential control, followed by rinsing the sample with ultrapure water using the shower tube. In the final step, the sample is transferred back to the load-lock chamber, and the gate valve is closed. Lastly, the load-lock chamber is evacuated, and the sample is transferred back into the main UHV chamber for further characterisation, including NAP-XPS, LEED and STM/nc-AFM.

OER onset estimation

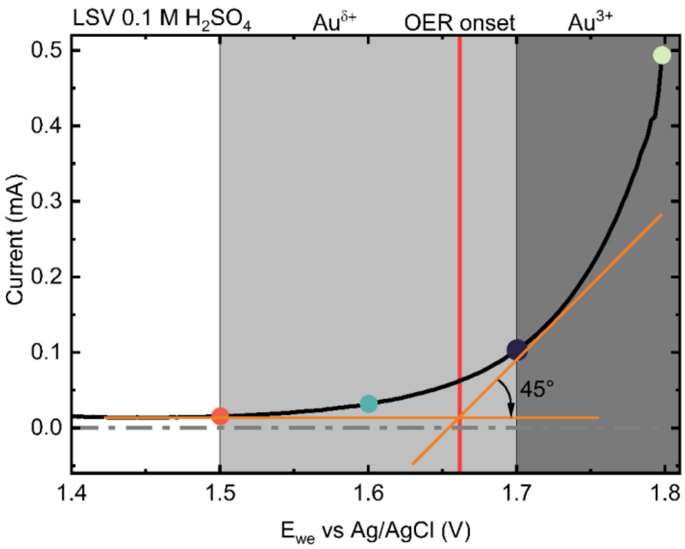

Supplementary figure 2. Detail of the LSV shown in Figure 1a. The onset of the OER is estimated at the intersection between the current baseline, and the tangent to the LSV curve at a slope of 45° [6]. The onset potential of OER is 1.66 V vs Ag/AgCl.

Cyclic voltammetry (CV) conditioning

The sample is brought into contact at -0.1 V and then immediately CV sweeping starts between -0.1 and the potential of the planned chronoamperometry (CA) experiment (1.5–1.8 V). Five CVs, figure S2, are swept before a final LSV between -0.1 and the target CA experiment. After the LSV the CA experiment starts for 5, 30 or 60 minutes. The contact withthe electrolyte is broken at the applied CA potential. The sample is not set to OCP between these steps.

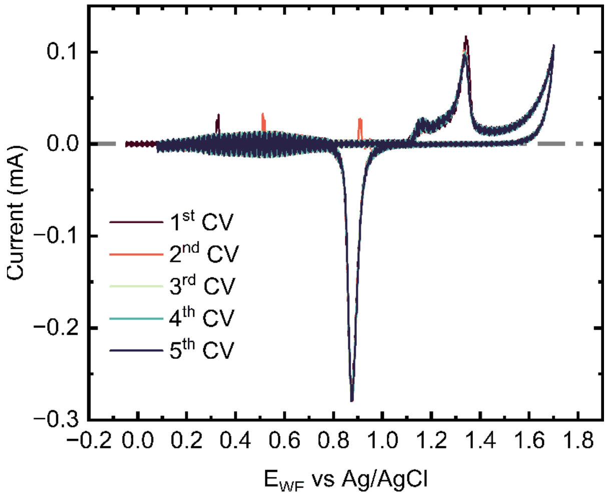

Supplementary figure 3. CV conditioning of Au(111) after UHV sputter/annealing cleaning. Five consecutive CVs are performed before the final LSV and CA experiments to obtain equivalent electrochemically conditioned surfaces across different samples.

Au 4f spectra of different emersion experiments after 30-min CA at 1.7 V in 0.1 M H_2SO_4

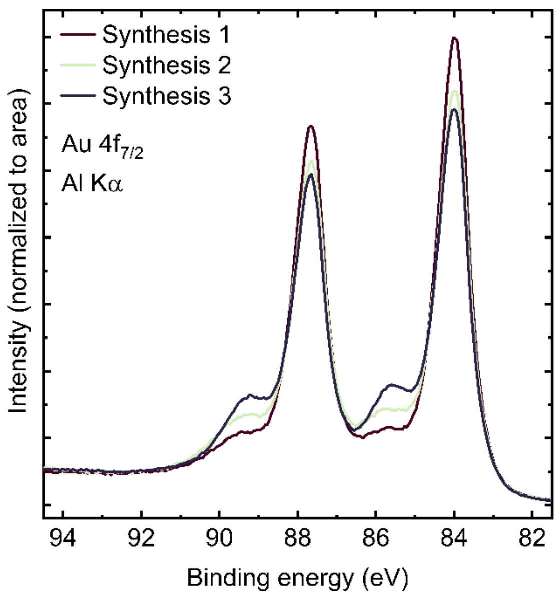

Supplementary figure 4. Au 4f signal of three different Au(111) samples emersed after 30-min CA at 1.7 V.

Estimation of amount of impurities

The electrooxidation of Au(111) occurs at high anodic potentials where carbon species are oxidized into CO_2. In addition to the Au 4f and O 1s spectra, we acquire the C 1s and S 2p regions to track impurities or electrolyte residue after the experiments. Figure S5a and b show a comparison of the C 1s and S 2p obtained after 30 min CA at 1.7 V and on clean Au(111). C is found at 284 eV, which corresponds to C-C species, and we do not detect SO_x or S species from the electrolyte. Figure S5c shows a quantitative comparison of our C amount across different experiments. We employ the open-access available XPS data from clean Au(111) and 1 ML of DBBA molecules on Au(111) in [5]. The sensitivity difference between the cited word and our instruments is calibrated by comparing the published Au 4f spectra of clean Au(111) and our own spectra. We obtained an approximately 0.4–0.5 ML C coverage after synthesis (orange) and after 72 h reduction in UHV (dark blue). We note that annealing to 200 ºC (light blue) removes the C content.

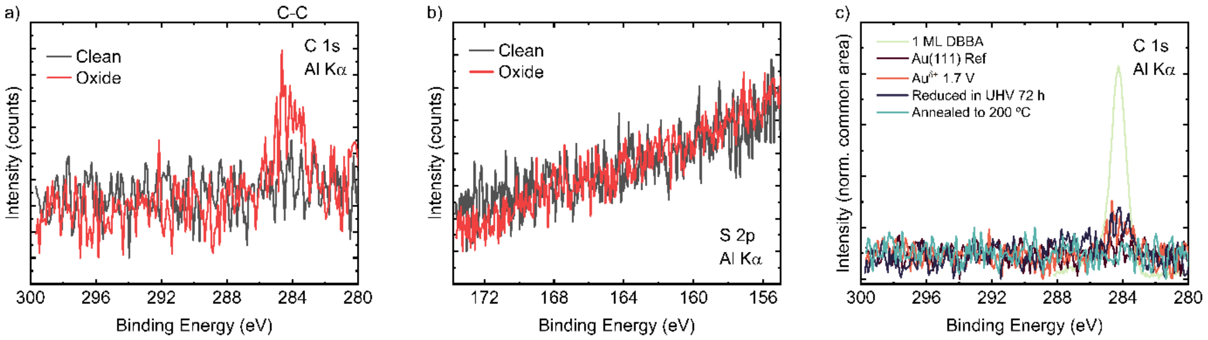

Supplementary figure 5. a) and b) comparison of the C and S content between a Au oxide sample emersed from the EC cell and clean Au(111). c) Comparison of the C 1s spectra of 1 ML of DBBA molecules[5] (light green) and clean Au(111) (purple), a Au oxide sample after 30 min CA at 1.7 V (orange), the oxide reduced in UHV for 72 h (dark blue) and the oxide reduced by 200 °C annealing (light blue).

STM after OCP and sample rinsing

The C amount on the surface does not increase after prolonged UHV exposure, Figure S5c dark blue spectrum, and C species are oxidized during CV and CA experiments. We propose that C impurities are brought upon the surface during rinsing of the electrolyte after the electrochemical experiments. Figure S6 shows an overview STM image of the surface after contact with the electrolyte in OCP and subsequent rinsing with MiliQ water. The surface features the characteristic herringbone reconstruction of Au(111) interrupted by dirt islands. The islands feature sharp edges aligned to the herringbone directions. The C coverage varies locally from 0.2 to 0.5 ML. Figure S6 shows a 200 × 200 nm^2 area with the largest coverage found across multiple overviews.

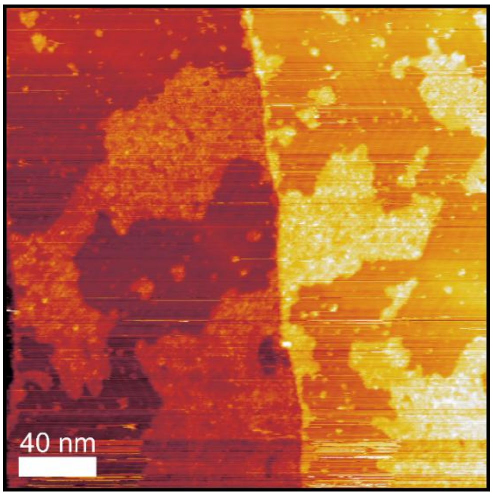

Supplementary figure 6. STM overview of the Au(111) surface after OCP wetting in 0.1 M H_2SO_4 and subsequent MiliQ water rinsing. V_{sample} = 2 V, $I_{setpoint}$ = 10 pA.

OCP Measurements of Au oxide

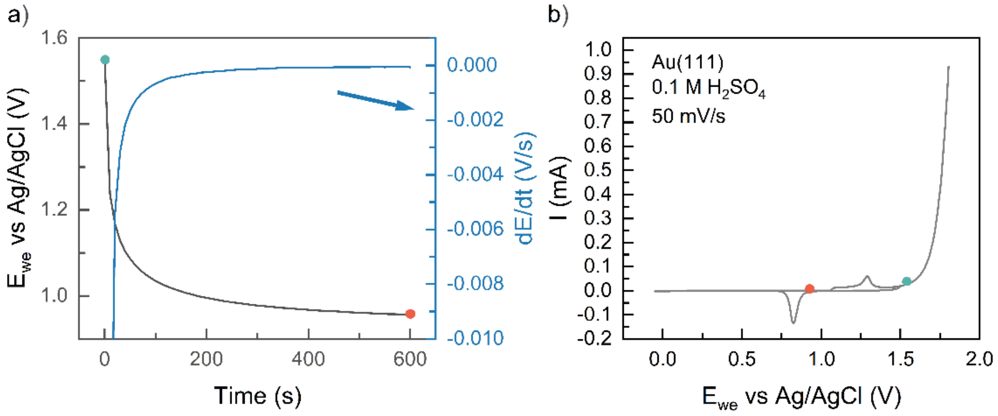

Supplementary figure 7. a) OCP decay and its derivative with time of an Au(111) electrode electrochemically oxidized at 1.8 V for 30 minutes in 0.1 M H_2SO_4. b) Cyclic voltammogram and linear sweep voltammetry of the Au(111) electrode, recorded before the oxidation at 1.8 V. The green dot on a) and b) marks the electrode's potential once the 1.8 V application is removed. The orange dot indicates the potential after 10 minutes at OCP, clearly above the potential of Au oxide reduction.

Clean Au(111) reference

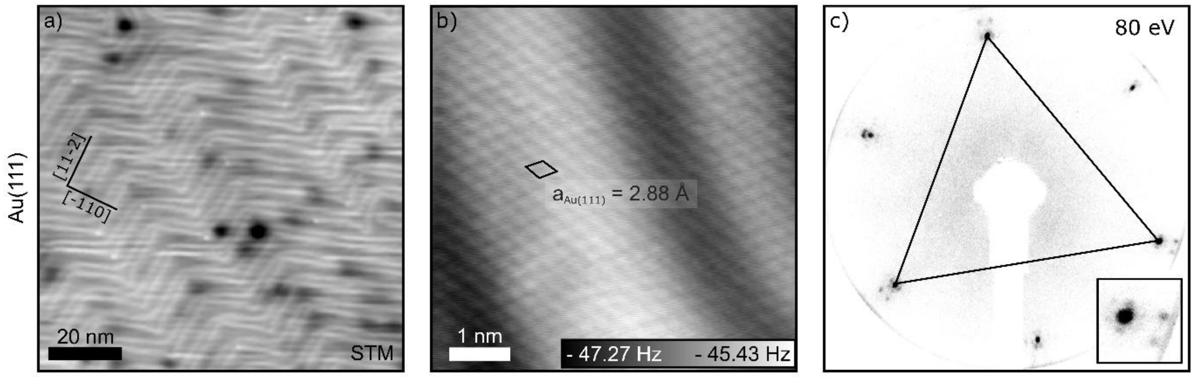

Supplementary figure 8. a)–c) UHV-prepared Au(111). a) STM image showing the characteristic herringbone reconstruction. Constant-current mode, V_{sample} = 0.4 V, $I_{setpoint}$ = 50 pA. b) Atomically-resolved view of Au(111). Constant-height mode, V_{sample} = 0.1 mV. c) LEED pattern of Au(111), the dark triangle marks the main spots of the trigonal (111) surface symmetry. The spots decorating the main spots reflect the herringbone reconstruction. The inset shows a detail of these spots.

Detail of clean Au area on $Au^{\delta+}$

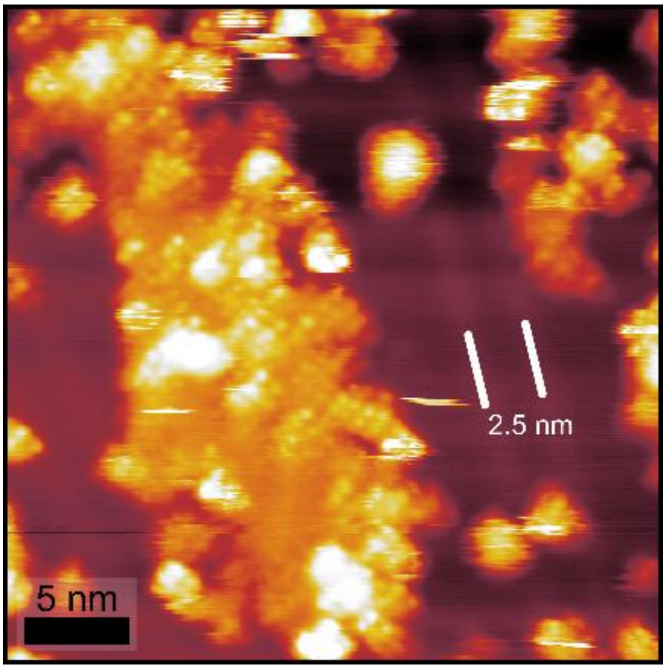

Supplementary figure 9. Detail of a clean Au(111) area next to a $Au^{\delta+}$ oxide island where a line structure reminiscent of the Au(111) herringbone reconstruction can be observed. V_{sample} = 1 V, $I_{setpoint}$ = 5 pA

Thick $Au^{\delta+}$ oxide

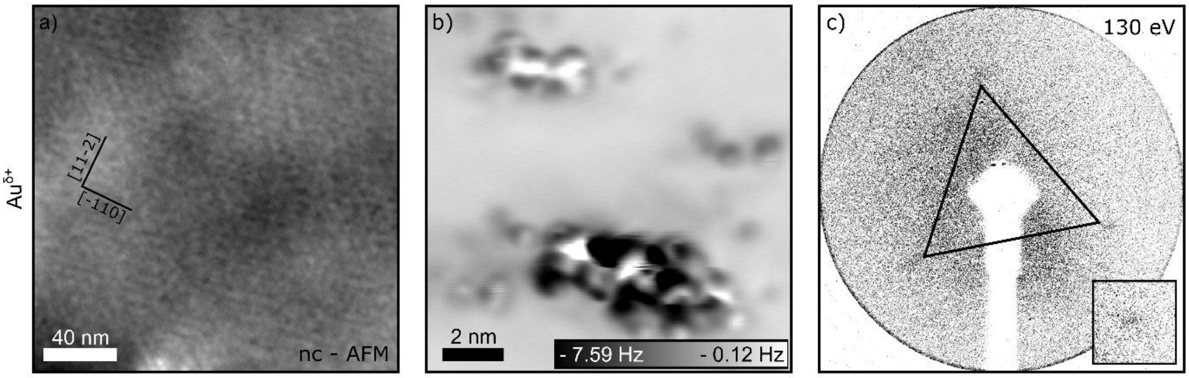

Supplementary figure 10. a)–c) $Au^{\delta+}$ surface produced by 30-minute CA at 1.7 V in 0.1 M H_2SO_4. d) nc-AFM image showing the cluster-decorated surface after CA. Constant-frequency shift mode, V_{bias} = 1.1 V, $\Delta f_{setpoint}$ = -3.24 Hz. e) close-up view of the clusters building up the $Au^{\delta+}$ oxide. Constant-height AFM mode, V_{sample} = 0.8 V, amplitude = 150 pm. f) Corresponding LEED pattern, only one set of three main spots are resolved at high electron beam energy. The inset shows a magnification of one of the spots.

Au$^{\delta+}$ reduction in UHV

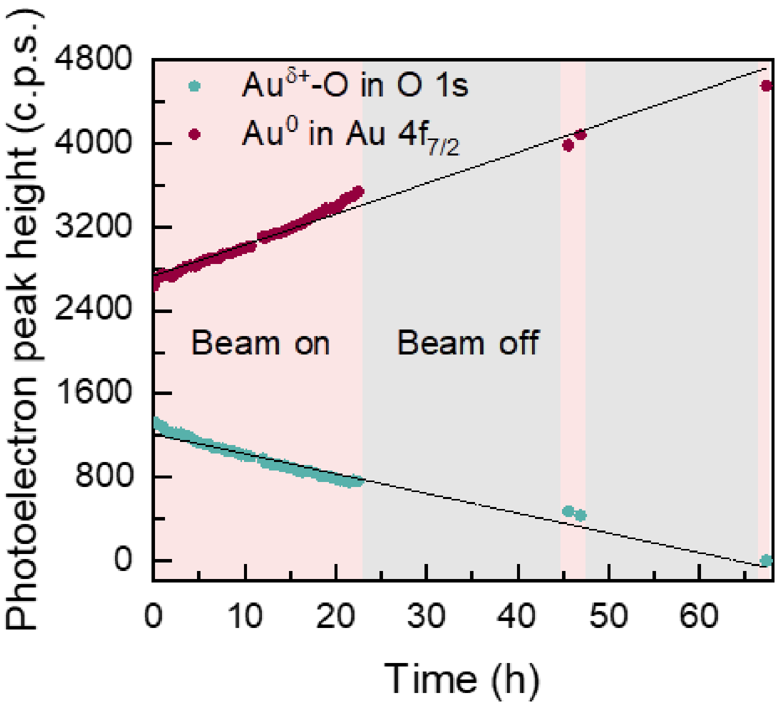

Supplementary figure 11. Evolution of the photoelectron peak height intensities for Au^0 in Au 4f$_{7/2}$ and Au$^{\delta+}$–O in O 1s of a Au(111) sample after 30 min CA at 1.7 V and left in UHV. Black lines represent linear fits. The light, red-shaded areas indicate periods of continuous beam exposure, while the grey-shaded areas correspond to when the beam was off.

Au^{3+} reduction in UHV vs 10 mbar H_2O

To test whether maintaining a water-vapor environment could prevent reduction of oxidized Au -thereby preserving hydrated species that might otherwise desorb under UHV, we performed in situ XPS under simultaneous X-ray illumination with near-ambient-pressure H_2O backfilling (10 mbar). Samples were transferred under 10 mbar H_2O and measured in situ under the same atmosphere. After oxidation at 2 V for 30 min, which yields a Au^{3+}-bluk surface oxide, the Au 4f signal attributable to Au^{3+} decays much faster under H_2O backfilling than in UHV, consistent with beam–water–accelerated reduction.

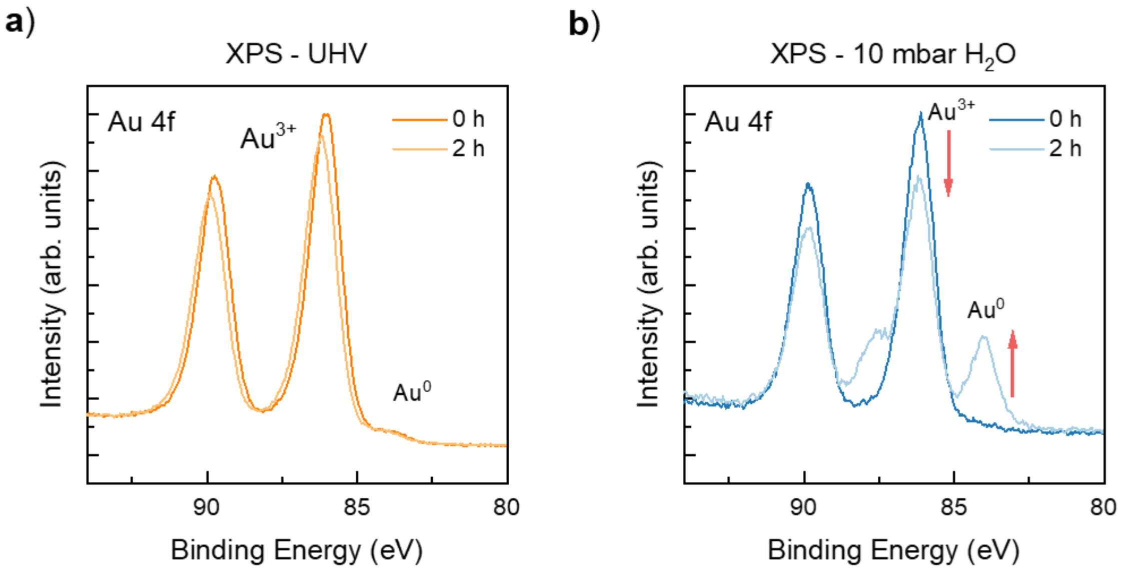

Supplementary figure 12. Au 4f XPS spectra of an Au(111) electrode electrochemically oxidized at 2 V for 30 minutes in 0.1 M H_2SO_4, measured initially and after continuous measurement over 2 hours under UHV a) and under 10 mbar H_2O vapor b).

Air exposure to $Au^{\delta+}$ oxide

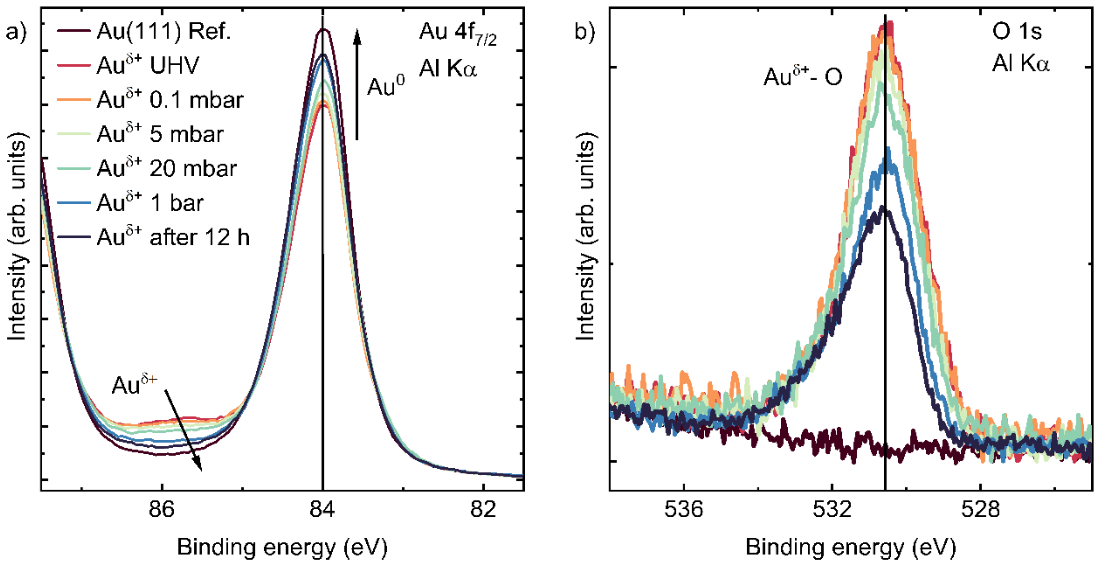

Supplementary figure 13. Evolution of the $Au^{\delta+}$ oxide after air exposure from 0.1 mbar to 1 bar. Air was dosed for 5 minutes without X-ray illumination. The spectra after 12 hours in UHV after 1 bar exposure is shown as a reference of UHV reduction.

LEED of $Au^{\delta+}$ oxide after thermal reduction

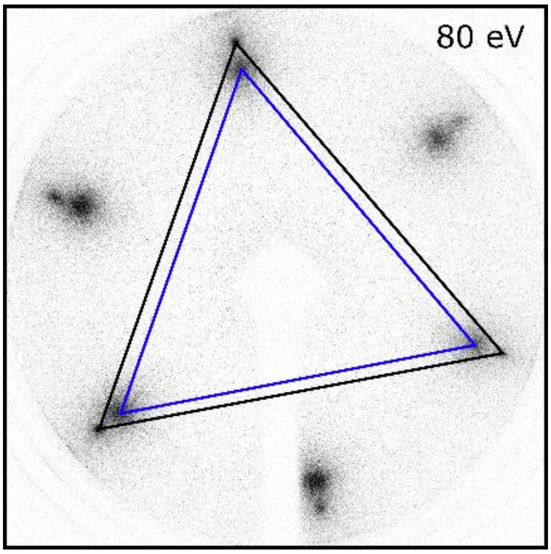

Supplementary figure 14. LEED pattern showing both the cluster and substrate periodicities, blue and dark triangles, respectively.

References

(1) Simanenko, A.; Samal, P. K.; Hübsch, R.; Škvára, J.; Yang, J.; Kastenmeier, M.; Winkler, F.; Skála, T.; Tsud, N.; Mehl, S.; Mysliveček, J.; Brummel, O.; Lykhach, Y.; Libuda, J. Origin of the Low Overpotential for Isopropanol Oxidation on Pt-Ru Electrocatalysts. *ACS Energy Lett* **2024**, *9* (10), 4875–4882. https://doi.org/10.1021/acsenergylett.4c01987.

(2) Samal, P. K.; Škvára, J.; Výhonský, M.; Fusek, L.; Ronovský, M.; Johánek, V.; Kastenmeier, M.; Lykhach, Y.; Libuda, J.; Brummel, O.; Mysliveček, J. Nanopatterning Single-Crystalline Metal Electrodes via Ion Erosion: New Structural Motifs for Model Electrocatalysis. *J Phys Chem Lett* **2025**, *16* (29), 7420–7427. https://doi.org/10.1021/acs.jpclett.5c01465.

(3) Sun, Z.; Lauritsen, J. V. A Versatile Electrochemical Cell for Hanging Meniscus or Flow Cell Measurement of Planar Model Electrodes Characterized with Scanning Tunneling Microscopy and X-Ray Photoelectron Spectroscopy. *Review of Scientific Instruments* **2021**, *92* (9). https://doi.org/10.1063/5.0060643.

(4) Brummel, O.; Bertram, M.; Prössl, C.; Ronovský, M.; Knöppel, J.; Matvija, P.; Fusek, L.; Skála, T.; Tsud, N.; Kastenmeier, M.; Matolín, V.; Mayrhofer, K. J. J.; Johánek, V.; Mysliveček, J.; Cherevko, S.; Lykhach, Y.; Libuda, J. Cobalt Oxide-Supported Pt Electrocatalysts: Intimate Correlation between Particle Size, Electronic Metal-Support Interaction and Stability. *Journal of Physical Chemistry Letters* **2020**, *11* (19), 8365–8371. https://doi.org/10.1021/acs.jpclett.0c02233.

(5) Lawrence, J.; Berdonces-Layunta, A.; Edalatmanesh, S.; Castro-Esteban, J.; Wang, T.; Jimenez-Martin, A.; de la Torre, B.; Castrillo-Bodero, R.; Angulo-Portugal, P.; Mohammed, M. S. G.; Matěj, A.; Vilas-Varela, M.; Schiller, F.; Corso, M.; Jelinek, P.; Peña, D.; de Oteyza, D. G. Circumventing the Stability Problems of Graphene Nanoribbon Zigzag Edges. *Nat Chem* **2022**, *14* (12), 1451–1458. https://doi.org/10.1038/s41557-022-01042-8.

(6) Yang, S.; Hetterscheid, D. G. H. Redefinition of the Active Species and the Mechanism of the Oxygen Evolution Reaction on Gold Oxide. *ACS Catal* **2020**, *10* (21), 12582–12589. https://doi.org/10.1021/acscatal.0c03548.
